# 752 Holding the Line: Decreasing CLABSI Rates in Burn Patients

**DOI:** 10.1093/jbcr/irae036.294

**Published:** 2024-04-17

**Authors:** Robertino Simpson, Brooke Dwars, Sara Wellsandt, Jolyn K Schneider, Matthew Slagel, Colette Galet, Lucy Wibbenmeyer

**Affiliations:** University of Iowa, Iowa City, IA; University of Iowa, Iowa City, IA; University of Iowa, Iowa City, IA; University of Iowa, Iowa City, IA; University of Iowa, Iowa City, IA; University of Iowa, Iowa City, IA; University of Iowa, Iowa City, IA

## Abstract

**Introduction:**

Burn patients are at higher risk of hospital-associated infections (HAI) secondary to loss of the protective property of skin. Hospitals systematically track central line blood stream infections (CLABSI) with a goal of zero infections. We noted an uptick in our CLABSI rates from 2017 to 2019. A quality improvement project (QI) was implemented to improve CLABSI rates.

**Methods:**

All burn patients 15 years and older admitted from 2017-2022 with a central line placed during their stay were included. The burn team instituted a multidisciplinary CLABSI intervention in three phases. The intervention began in 11/2019. It included education focused on occlusive dressings, protection from water during hydrotherapy, and daily discussion on the need for the central line. Prior to implementation, catheters were changed at the discretion of the team. Catheters were changed every 7 ± 2 days starting in 11/2019 (phase 1), every 7 days ± 1 day starting in 9/2020 (phase 2) and every 7 days in 3/2021 (phase 3). CLABSI rates were defined as number of infections per 1000 central line days. The pre-implementation period (1/2017 to 10/2019) served as the control. Demographics, burn injury, and central line data information were collected on patients who developed CLABSIs

**Results:**

CLABSI rate was 3.6% during the control period. It went up to 7.5% and 6.7% after phase 1 and 2, respectively, and decreased to 1.3% after phase 3 with no CLABSIs in 2022. The median number of central line days were 8 [4-14] overall, 11 [8-14] pre-implementation, 7 [4-8] post-phase 1 and 2, and 7 after phase 3. Patients who developed CLABSI were mostly male (80%) with a median age of 45.5 y [19-78]; 40% presented with diabetes, 30% with hypertension, and 20% were smokers. Median %TBSA was 54.5% [11%-88%], and 20% had inhalation injury. The median length of stay was 104 [11-273] days. A week prior to CLABSI, their average blood glucose level was 203 ± 51.5mg/dl. Candida and Pseudomonas accounted for most of the infections (n= 4 (40%) for each). Four patients developed CLABSI in winter, 3 in summer, 2 in spring, and one in fall.

**Conclusions:**

Increasing nurse education and strict 7-day central line replacement led to a decrease in the CLABSI rates in burn patients with zero infections in phase three. There was a spike in blood glucose levels the week prior to CLABSI documentation. The relationship between blood glucose control and infections requires more investigation

**Applicability of Research to Practice:**

CLABSI rates can be reduced by nurse-led education and strict adherence to a 7-day central line replacement

